# Intra-Specific Regulatory Variation in *Drosophila pseudoobscura*

**DOI:** 10.1371/journal.pone.0083547

**Published:** 2013-12-27

**Authors:** Anton Suvorov, Viola Nolte, Ram Vinay Pandey, Susanne U. Franssen, Andreas Futschik, Christian Schlötterer

**Affiliations:** 1 Institut für Populationsgenetik, Vetmeduni Vienna, Vienna, Austria; 2 Vienna Graduate School of Population Genetics, Vienna, Austria; 3 Department of Applied Statistics, Johannes Kepler Universität Linz, Linz, Austria; University of Oxford, United Kingdom

## Abstract

It is generally accepted that gene regulation serves an important role in determining the phenotype. To shed light on the evolutionary forces operating on gene regulation, previous studies mainly focused on the expression differences between species and their inter-specific hybrids. Here, we use RNA-Seq to study the intra-specific distribution of *cis*- and *trans*-regulatory variation in *Drosophila pseudoobscura*. Consistent with previous results, we find almost twice as many genes (26%) with significant *trans*-effects than genes with significant *cis*-effects (18%). While this result supports the previous suggestion of a larger mutational target of *trans*-effects, we also show that *trans*-effects may be subjected to purifying selection. Our results underline the importance of intra-specific analyses for the understanding of the evolution of gene expression.

## Introduction

It is well understood that variation in gene expression is an important source of phenotypic differences. Hence, it has been a long-term goal in biology to understand the regulation of gene expression. The genetic basis of variation in gene expression can be divided into two classes: 1) Variation in regulatory domains (*cis*-regulatory elements) that modulates gene expression or polymorphisms that influence the stability of mRNA. 2) Variation in *trans*-acting proteins, e.g. transcription factors that regulate the expression of a set of target genes. The combined effects of *cis*- and *trans*-regulation determine the expression of every gene. Different approaches ranging from diallel crosses [1] to eQTLs [2] have been pursued to understand the regulation of gene expression, but the highest level of resolution has been obtained from allele-specific gene expression measurements in parents and their offspring. Since offspring share the *trans*-acting factors from both parents the contrast of gene expression in parents and offspring provides an estimate for the magnitude of *cis*- and *trans*-effects [3].

The contribution of *cis*- and *trans*-effects to the evolution of gene expression has been studied in several species (e.g. in *Drosophila*: [3], [4], [5], [6], yeast: [7], [8], *Arabidopsis*: [9]). Independent of the species studied, frequently more *cis*- than *trans*-effects were found when two closely related species and their hybrids were analyzed. This observation is particularly interesting since the mutational target of *trans*-effects (i.e.: all *trans*-regulating factors) is larger than the one of *cis*-effects [10], [11]. The interpretation of the greater amounts of *cis-*effects found is that *trans*-effects are more pleiotropic than *cis*-effects. Thus, *trans*-mutations are more difficult to establish and become fixed even if they have a beneficial effect on one target gene as other target genes can be negatively affected by the mutation. In contrast, *cis*-mutations affect a single gene only. Therefore, they tend to be less deleterious than *trans*-effects and are more likely to become fixed [4].

The comparison of mutation accumulation lines, with very low selection efficacy due to a small effective population size, to natural isolates also showed that *trans*-effects are subject to strong purifying selection [11]. Furthermore, *D. sechellia*, a species with a very small effective population size (and thus a lower selection efficacy) shows more *trans*-effects than the cosmopolitan *D. simulans* [6], [12], [13].

For intra-specific studies, the relative importance of *cis*- and *trans*-effects is still controversial. Early attempts to understand the contribution of *cis*- and *trans*-regulatory components within *Drosophila* species showed more *trans*- than *cis*-effects in *D. simulans* males [14]. A study in *D. melanogaster* identified 90% *cis*-effects and only very few *trans*-effects, but it was not clear to what extent this observation results from low power to detect *trans*-effects [5]. A comparison of the two *D. melanogaster* strains with strong assortative mating, Z and M, suggested that the expression differences between them could be largely attributed to *cis*-regulatory variation [15]. In yeast, *trans*-effects were found to contribute more to intra-specific expression variation [7]. Among 863 of differentially expressed genes 558 (∼64%) had been influenced by *trans*-regulation, whereas 421 (∼49%) genes were found to have significant *cis*- regulation [7]. In *Arabidopsis thaliana*, more pure *trans*-effects than pure *cis*-effects were found [16], which contrasts with an earlier study that found an excess of *cis*-effects in *A. thaliana* [1].

Since it is not clear to what extent the contrasting observations reflect differences in the experimental design and applied technologies, we studied the relative contribution of *cis*- and *trans*-effects to the intra-specific expression variation in *D. pseudoobscura*. Using the latest next generation sequencing technology we show that *trans*-effects are more abundant than *cis*-effects, probably reflecting their larger mutational target size. Furthermore, we show that the distribution of effect sizes differs between *cis*- and *trans*-effects, most likely caused by different selective forces operating on them.

## Materials and Methods

### Sample preparation and sequencing

The *D. pseudoobscura* strains ps94 (stock number 14011-0121.94) and ps88 (stock number 14011-0121.88) were obtained from the UC San Diego *Drosophila* Species Stock Center. Libraries of the parental strains were those used by Palmieri et al. [17]. Reciprocal hybrids were reared in parallel and under the same conditions as the parental strains by either crossing a virgin ps94 female with a ps88 male or a virgin ps88 female with a ps94 male. Flies were reared on standard cornmeal-molasse-yeast-agar medium and maintained at 19°C under constant dark conditions. Virgin female offspring of 15–20 replicate crosses from either direction were collected, allowed to age for three to seven days and shock-frozen in liquid nitrogen. For extraction of total RNA, we used equal numbers of females from the two directions of crosses, and performed two replicate RNA extractions that were pooled for library construction. This strategy aimed to reduce possible imprinting effects and the biological variation among crosses. Paired-end Illumina mRNA libraries were generated from 10 ug total RNA using the mRNA Sample Prep Kit (Illumina, San Diego, CA) as previously described [17]. All libraries were sequenced on the same flow cell as the female libraries of the two parental strains used in [17]. We did not sequence replicate libraries. The RNA-Seq read libraries were deposited in the ArrayExpress database https://www.ebi.ac.uk/arrayexpress/under the accession number E-MTAB-1424.

### Read mapping

Reads were trimmed using the Mott algorithm implemented in PoPoolation [18] (minimum read length = 40, quality threshold = 20). We mapped three RNA-Seq paired-end (2×100 bp) libraries derived from ps88 females, ps94 females and hybrid females, against the *D. pseudoobscura* reference genome (FlyBase, assembly r2.23) using GSNAP [19]. Only non-ambiguously mapping read pairs were retained. To minimize the mapping bias, we first mapped the reads from the parental lines to the reference and identified SNPs fixed between the two strains. Then, we remapped the reads including the SNP information with GSNAP. Using an improved *D. pseudoobscura* annotation [17] we identified SNPs that were fixed (allele frequency = 1) for different alleles in the two parental lines. Based on these SNPs we generated two distinct reference genomes, one for each parental strain. Allele specific gene expression was measured by mapping the RNA-Seq reads to both genomes simultaneously and counting the number of reads mapping unambiguously to one of the two genomes (i.e. read pairs which covered at least one of the SNPs distinguishing the two genomes). The generation of two parental genomes not only identifies reads from either parental allele but also aims to avoid a well described mapping bias for different allelelic variants [20], [21]. Moreover, it has been shown that after the adjustment for the different parental alleles via two reference genomes a mapping bias can remain for some genes [21]. To quantify and account for this remaining mapping bias we determined an empirical correction factor through simulations. These simulations included the following steps: 1) For each gene the same number of RNA-Seq reads (paired ends) were generated for the identical positions in the two genomes matching the read length and insert size of the empirical data. (i.e.: differing only by the identified SNPs). 2) The simulated RNA-Seq read libraries were mapped against both genomes simultaneously retaining only non-ambiguously mapped reads. 3) For genes with a different number of reads mapping to each of the genomes, we determined a correction factor, which is the inverse of the ratio of the mapped reads. This correction factor was then included in the G-test to correct the Null-expectation for allele specific expression in hybrids.

Finally, we normalized the expression data using the TMM method [22]. The pipeline described above to identify allele-specific gene expression analysis is implemented in the Allim software package (http://code.google.com/p/allim/, [21]).

### Data analysis

*Cis*- and *trans*-effects were estimated by contrasting gene expression in the parental and F1 libraries. In order to test for significance of the regulatory scenarios we used the G-test of independence followed by correction using the Benjamini-Hochberg method at a false discovery rate cutoff (FDR) of 0.05. Testing was performed in a gene-wise manner. Total differential expression (TDE) between parents was estimated by comparing the ratio of the counts in the two parents to the ratio of total counts (i.e.: ratio of library sizes). For allele specific differential expression in F1 individuals (*cis*), we applied the same logic to the F1 library and compared the ratio of allelic counts at a given gene to the ratio of the allele counts summed over all genes. For the *trans*-test, we compared the ratio of parental gene expression counts to the ratio of allele specific counts in the F1 library. We note that this testing strategy results in a lower power to detect differences in *trans* compared to *cis*-effects since the total number of counts is higher in our tests for *cis*-effects than in our tests for *trans*-effects (see below). Following previous studies [6], [23], we distinguish between different types of regulation namely *cis*, *trans*, *cis*-by-t*rans*, *cis*+*trans*, compensatory, conserved and ambiguous (Table S1). Modes of inheritance were classified as additive, dominant, over-dominant, under-dominant and conserved expression as described by Landry *et al*. [23] and McManus *et al*. [6] (Table S2).

### Statistical power estimation for *cis*- and *trans*-tests through simulations

We hypothesized that statistical power of the G-test for allele-specific differential expression (*cis*) is higher than for *trans*-test due to the fact that only one “noisy” allele-specific expression measurements were included into the *cis*-tests whereas two such measurements appeared in a *trans*-test. To support this hypothesis we performed computer simulations, in which we compared the power for different effect sizes. Since the power critically depends on the expression level of a given gene, we based our simulations on the observed expression level *n* (averaged over the three libraries) of randomly selected genes. The allelic imbalance *γ* was modulated on a grid of values ranging from 1 to 10 with the step size of 0.2. We simulated allele expression counts taken from a Poisson (*λ*) distribution for the parental lines (P1 and P2) and from a Poisson (*λ*/2) distribution for the hybrid lines (H1 and H2). For each gene two different values *λ*_1_ and *λ*_2_ were computed according to *λ*_1_ = 2*n*/(*γ*+1), *λ*_2_ = 2*n γ*/(*γ*+1). For pure *cis*-effects we simulated expression counts from H1 according to Poisson (*λ*_1_/2) and for H2 according to Poisson (*λ*_2_/2), and Poisson (*λ*_1_) for P1 and Poisson (*λ*_2_) for P2. For pure *trans*-effects, we took Poisson (*λ*_1_) for P1 and Poisson (*λ*_2_) for P2, and Poisson (*n*/2) for H1 and H2. Combined *cis*- and *trans*-effects can be simulated by taking *λ*_P1_ = 2*n*/(*γ*_P_+1), *λ*_P2_ = 2*n γ*_P_/(*γ*_P_+1), *λ*_H1_ = 2*n*/(*γ*_H_+1) and *λ*_H2_ = 2*n γ*_H_/(*γ*_H_+1) with allelic imbalances *γ*_P_ and *γ*_H_ for parents and hybrids. Using the proportion of rejections, the statistical power (1-Type II error) of the tests was estimated by simulating expression counts for 10000 genes under each imbalance parameter value (*γ*, and (*γ*_P_, *γ*_H_)). All statistical analyses were performed using R.

### GO analysis

GO analysis were performed with FuncAssociate 2.0 [24] using the gene IDs of the *D. melanogaster* orthologs of *D. pseudoobscura*.

## Results

We performed RNA-Seq for two highly inbred parental lines (ps88 and ps94) and F1 individuals obtained from bi-directional crosses of the two inbred lines. On average, we obtained about 41×10^6^ reads for each library (Table S3), which resulted on average in about 4×10^6^ unambiguously mapping read pairs for each library after all filtering steps. Out of 16743 annotated genes in *D. pseudoobscura* [17], we identified 8116 (48.5%) genes with at least one fixed difference between the two lines. On average, we detected 4.4 fixed SNPs per gene between the two lines. 7631 genes with more than 20 reads in both parental lines (ps88+ps94≥20) were included in the subsequent analysis.

### Classification of *cis*- and *trans*-regulation

Gene expression is determined by *cis*- and *trans*-regulatory variation. Since variation in *cis*-regulatory elements results in allele specific gene expression, it is possible to measure *cis*-effects in F1 individuals. We detected significant (FDR≤0.05) differences in gene expression due to *cis*-effects between the two parental alleles for 1359 genes (including 154 genes from the ambiguous category, Table S1), which correspond to 18% of the expressed genes. *Trans*-effects were estimated by a significant (FDR≤0.05) difference in gene expression for the same allele in the parental and F1 background [3], [4], [5], [6]. We identified 1982 (26%) genes (including 89 genes from the ambiguous category, Table S1) with significant *trans*-regulation.

**Figure 1:**
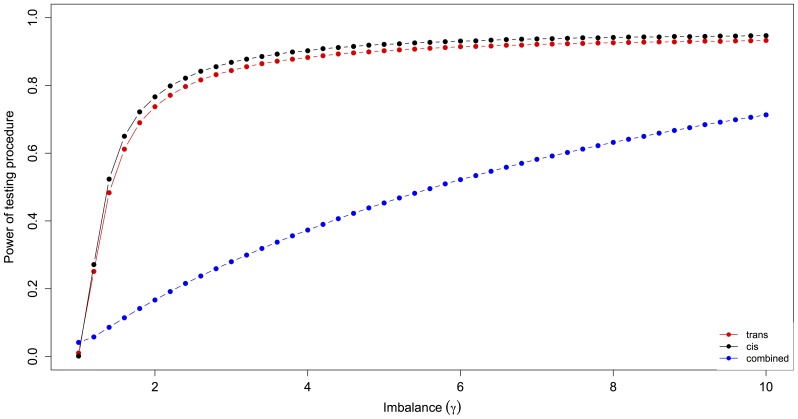
Power analysis of *cis*- and *trans*-tests. We used computer simulations to determine the power to detect *cis*-effects (black), pure *trans*-effects (red), and *trans*-effects in genes with combined regulatory modes (blue). The power was determined for 10000 randomly selected genes over a range of imbalance indices (*γ*) and the averages are shown. The imbalance indices for genes with combined effects were calculated as |*γ*_P_ – *γ*_H_|+1).

**Figure 2:**
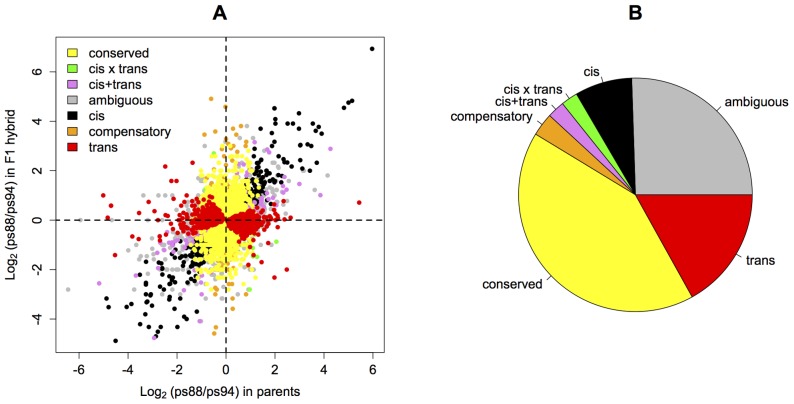
*Cis-* and *trans*-regulatory differences. A) Distribution of regulatory effects depending on parental expression and allele specific expression in the hybrid. B) Relative distribution of the regulatory types (*cis*: 604 (7.9%); *trans*: 1292 (16.9%); *cis*–by–*trans*: 176 (2.3%); *cis*+*trans*: 183 (2.4%); compensatory: 242 (3.2%); conserved: 3187 (41.8%); ambiguous: 1947 (25.5%)).

Following the classification introduced by [23] we grouped the expressed genes in further sub-categories (Table S4). We identified 604 genes for which only *cis*-regulation was significant. More than twice as many genes (1292) had only significant *trans*-effects. We used computer simulations to test if the large excess of genes with *trans*-effects could be an artifact of our method. Interestingly, our simulations showed a slightly lower power to identify *trans*-effects over a wide range of imbalances (Fig. 1). For genes with combined *cis*- and *trans*-effects the power of the testing procedure was substantially reduced on average, as shown by the total area under the power curve that is smaller for combined effects than for pure *cis* and *trans* effects (Fig. 1). This lower power to detect *trans*- than *cis*-effects makes the observed excess of genes with *trans*-effects particularly relevant since it is most likely an underestimate. For 601 genes we identified combined *cis*- and *trans*-effects: *cis*-by-*trans* (176), *cis*+*trans* (183) and compensatory (242). For 3187 (42%) genes the gene expression pattern was conserved among the three samples. The remaining 1947 genes had an ambiguous expression pattern with no clear biological interpretation, i.e. the genes that cannot be placed in any category of regulation based on their patterns of parental and hybrid expression (Figure 2A,B). Using more stringent significance FDR thresholds of 0.01 and 0.005 did not change the overall pattern (Figure S1).

**Figure 3:**
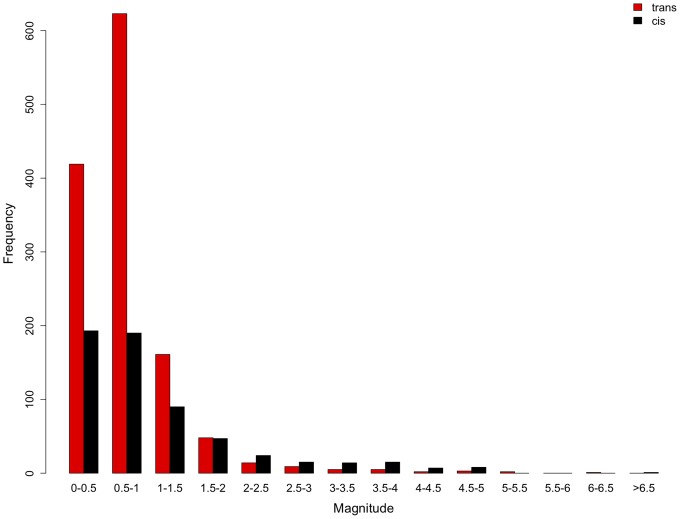
Frequency distribution of genes with significant *cis*- and *trans*- regulation. Genes with pure *trans* (red) and *cis* (black) effects were grouped according to the magnitude of their effect.

Our observation of more genes with *trans*-effects than *cis*-effects is consistent with the larger mutational target of *trans*-effects. To further scrutinize this hypothesis we determined the frequency distribution of *cis*- and *trans*-regulatory effect sizes (*cis*-magnitude =  |log2(ps88 hybrid/ps94 hybrid)|, *trans*-magnitude =  |log2(ps88/ps94) – log2(ps88 hybrid/ps94 hybrid)| and noticed a striking difference. In the small effect size class we detected a significant excess of genes with *trans*-effect. In all classes with a larger effect size, we found an excess of genes with *cis*-effects (Figure 3). Qualitatively the same result was obtained when genes with combined effects were also included (data not shown). This marked difference between effect size classes is consistent with stronger purifying selection operating on genes with *trans*-effects, possibly due to their pleiotropic nature.

### Dissecting modes of inheritance

**Figure 4:**
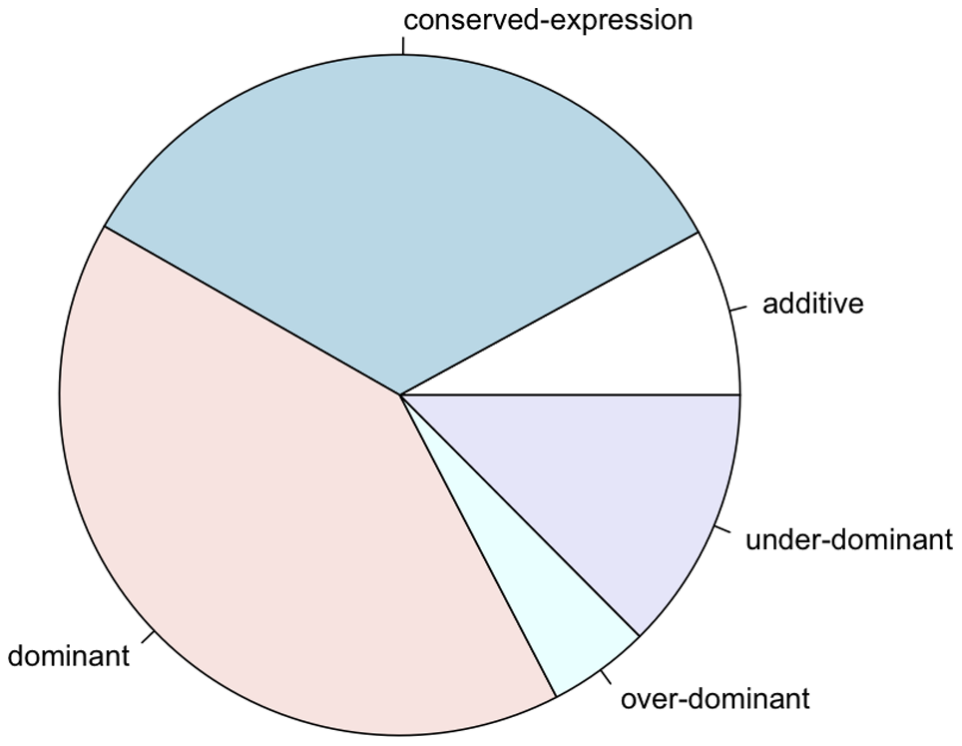
Modes of inheritance. Relative proportions of the inheritance modes (dominant: 3120 (40.9%); additive: 607 (8%); over-dominant: 369 (4.8%); under-dominant: 957 (12.5%); conserved-expression: 2578 (33.8%)).

The comparison of expression intensities in F1 individuals relative to the one in their parents provides information about the mode of inheritance (Table S2). Out of 7631 genes analyzed, 607 genes were classified as additive and 3120 genes as dominant. 1326 genes were misexpressed in the F1 individuals, with 369 genes being over-dominant (higher expression in the F1) and 957 genes being under-dominant (lower expression in the F1 than in both parents). 2578 genes had no significant difference between parents and the F1 (conserved, Figure 4). We note, that the high fraction of genes with conserved inheritance reflects statistical power of our experiment rather than a true biological signal since the genes with conserved mode of inheritance have lower expression intensities (ps88 parent+ps94 parent+F1) than those genes with additive, dominant or misexpressed modes of inheritance (Wilcoxon's one-tailed rank-sum test, P<2.2e-16). As expected, we found no indication of one parent being over-represented among the genes with a dominant mode of inheritance: among 3 120 genes 1518 (∼49%) and 1602 (∼51%) genes have ps88-like and ps94-like expression.

Intra-specific comparisons suggested that *cis*-regulation tends towards additivity whereas *trans*-regulation shows higher degrees of dominance [25]. Consistent with these previous results, we found that *cis*-magnitude (|log2(ps88 hybrid/ps94 hybrid)|) within genes with additive mode of inheritance was significantly greater than the *cis*-magnitude of genes with other significant inheritance types (Wilcoxon's one-tailed rank-sum test, P = 0.007738) (Figure S2).

### GO analysis

We tested for an enrichment of functional categories among the different modes of gene regulation using a gene ontology (GO) analysis. While some marginal significance was detected for some modes of gene regulation, none of them survived a correction for multiple testing. Similarly, no significant enrichment of a GO category was observed for the different modes of inheritance. Hence, we conclude that our data do not support a functional differentiation among the categories studied.

## Discussion

This report is the first study of intra-specific *cis*-and *trans*-effects using RNA-Seq in *Drosophila* and the first report for *D. pseudoobscura*. We observe about twice as many genes with pure *trans*-effects (1292) as genes with pure *cis*-effects (604). This result is consistent with earlier studies in *Drosophila* [4], *Arabidopsis* [16] and yeast [7]. Hence, we suggest that this pattern is most likely general and contrasting results [5] probably reflect more methodological differences than an alternative biological phenomenon.

Of particular interest is the contrast to studies of *cis*- and *trans*-effects relying on the analysis of expression differences between closely related species and their corresponding hybrids. These analyses typically observed a clear excess of genes with *cis*-effects [4], [8], [9], [26]. The prevailing explanation for this apparent discrepancy is that *cis*-effects affect only a single gene, while *trans*-effects are typically pleiotropic [3], [4], [27]. Hence, *cis*-effects become more easily fixed between species [28], [29]. Within species, however, the larger mutational target of *trans*-effects results in the observed excess of *trans*-effects [4], [7]. In our report, we have further scrutinized the hypothesis of differential selection operating on *cis*- and *trans*-effects by contrasting the frequency distribution of different effect sizes. Reasoning that *trans*-effect mutations with a large effect size are more effectively purged than *cis*-effect ones, we predicted an underrepresentation of genes with *trans*-effects in the large effect size classes. In fact, our data show a trend of a more pronounced underrepresentation of genes with *trans*-effects with an increasing effect size (Figure 3). Furthermore, the larger number of genes with *trans*-effects is consistent with them being a larger mutational target. Alternatively, the observed distribution of *cis*- and *trans*-effects (Figure 3) could be also obtained if new *trans*-mutations have, in general, smaller regulatory effect sizes than mutations in regulatory regions.

Overall, we conclude that the patterns of intra-specific and inter-specific *cis*- and *trans*-effects discussed above are compatible with the possibility that most regulatory variation is deleterious and their distribution reflects the balance between the occurrence of new mutations and their different fixation probabilities. This idea is in line with other results showing that purifying selection is the major force affecting the evolution of gene expression [30]. Nevertheless, if purifying selection shapes the pattern of *trans*-effects while positive selection contributes largely to *cis*-effects [31], the distribution of intra-specific *cis*- and *trans*-effect sizes may also differ.

We caution that the evolution of gene expression is probably more complex. One indication for this could be obtained from the comparison of the effect sizes in intra- and inter-specific comparisons. While we found an underrepresentation of genes with large *trans*-effects, inter-specific comparisons suggest large effect sizes of *trans*-effects [6]. We hypothesize that this discrepancy could be due to species specific *trans*-regulatory mutations that may, for example, be involved in compensatory changes.

To obtain a complete picture of the evolutionary dynamics of *cis*- and *trans*-effects it is important to analyze their context specific effects. Depending on the developmental stage, tissue, and environment the regulatory landscape may differ and consequently also the effects of *cis*- and *trans*-regulatory variation. More experiments are needed to understand to what extent the patterns described for a single sex, developmental stage and environment can be generalized. Furthermore, future studies may benefit from replication, which will improve the accuracy of the estimated effects beyond the already high repeatability of RNA-Seq studies [32].

## Supporting Information

Figure S1 **Effect of different FDR threshold on the identification of regulatory effects.** A) FDR < 0.05. B) FDR < 0.01. C) FDR <0.005.
(PDF)

Figure S2 ***Cis*** **-magnitude comparison of genes with additive and non-additive modes of inheritance.** Genes with additive mode (A) of inheritance exhibit higher *cis*-magnitude vs. all other genes with non-additive modes (NA) excluding genes with conserved expression mode.
(PDF)

Table S1 Determination of regulatory categories based on total differential expression between parents (TDE), allele-specific differential expression in F1 hybrid (*cis*) and *trans*-test. All the statistical tests were performed using G-test, followed by FDR correction (q≤0.05).
(DOC)

Table S2 Identification of inheritance modes based on differential expression and directionality of expression between parental and total expression in F1 hybrids.
(DOC)

Table S3 Library size information.
(DOC)

Table S4 Gene summary table. This table provides information about raw and normalized expression counts for parents and hybrids, statistical tests, regulatory types, inheritance modes and *cis* and *trans* magnitudes for each gene revealed by RNA-seq.
(XLS)

## References

[1] Kiekens R, Vercauteren A, Moerkerke B, Goetghebeur E, Van Den Daele H, et al (2006) Genome-wide screening for cis-regulatory variation using a classical diallel crossing scheme. Nucleic Acids Res 34: 3677–3686. 16885241 10.1093/nar/gkl510 PMC1540733

[2] Doss S, Schadt EE, Drake TA, Lusis AJ (2005) *Cis*-acting expression quantitative trait loci in mice. Genome Res 15: 681–691. 15837804 10.1101/gr.3216905 PMC1088296

[3] Wittkopp PJ, Haerum BK, Clark AG (2004) Evolutionary changes in *cis* and *trans* gene regulation. Nature 430: 85–88. 15229602 10.1038/nature02698

[4] Wittkopp PJ, Haerum BK, Clark AG (2008) Regulatory changes underlying expression differences within and between *Drosophila* species. Nat Genet 40: 346–350. 18278046 10.1038/ng.77

[5] Genissel A, McIntyre LM, Wayne ML, Nuzhdin SV (2008) *Cis* and *trans* regulatory effects contribute to natural variation in transcriptome of *Drosophila melanogaster* . Mol Biol Evol 25: 101–110. 17998255 10.1093/molbev/msm247

[6] McManus CJ, Coolon JD, Duff MO, Eipper-Mains J, Graveley BR, et al (2010) Regulatory divergence in *Drosophila* revealed by mRNA-seq. Genome Res 20: 816–825. 20354124 10.1101/gr.102491.109 PMC2877578

[7] Emerson JJ, Hsieh LC, Sung HM, Wang TY, Huang CJ, et al (2010) Natural selection on *cis* and *trans* regulation in yeasts. Genome Res 20: 826–836. 20445163 10.1101/gr.101576.109 PMC2877579

[8] Tirosh I, Reikhav S, Levy AA, Barkai N (2009) A yeast hybrid provides insight into the evolution of gene expression regulation. Science 324: 659–662. 19407207 10.1126/science.1169766

[9] Shi X, Ng DW, Zhang C, Comai L, Ye W, et al (2012) *Cis*- and *trans*-regulatory divergence between progenitor species determines gene-expression novelty in *Arabidopsis* allopolyploids. Nat Commun 3: 950. 22805557 10.1038/ncomms1954

[10] Landry CR, Lemos B, Rifkin SA, Dickinson WJ, Hartl DL (2007) Genetic properties influencing the evolvability of gene expression. Science 317: 118–121. 17525304 10.1126/science.1140247

[11] Denver DR, Morris K, Streelman JT, Kim SK, Lynch M, et al (2005) The transcriptional consequences of mutation and natural selection in *Caenorhabditis elegans* . Nat Genet 37: 544–548. 15852004 10.1038/ng1554

[12] Kliman RM, Andolfatto P, Coyne JA, Depaulis F, Kreitman M, et al (2000) The population genetics of the origin and divergence of the *Drosophila simulans* complex species. Genetics 156: 1913–1931. 11102384 10.1093/genetics/156.4.1913 PMC1461354

[13] Legrand D, Tenaillon MI, Matyot P, Gerlach J, Lachaise D, et al (2009) Species-wide genetic variation and demographic history of *Drosophila sechellia*, a species lacking population structure. Genetics 182: 1197–1206. 19506309 10.1534/genetics.108.092080 PMC2728859

[14] Wayne ML, Pan YJ, Nuzhdin SV, McIntyre LM (2004) Additivity and *trans*-acting effects on gene expression in male *Drosophila simulans* . Genetics 168: 1413–1420. 15579694 10.1534/genetics.104.030973 PMC1448806

[15] Osada N, Kohn MH, Wu CI (2006) Genomic inferences of the *cis*-regulatory nucleotide polymorphisms underlying gene expression differences between *Drosophila melanogaster* mating races. Mol Biol Evol 23: 1585–1591. 16754642 10.1093/molbev/msl023

[16] Zhang X, Borevitz JO (2009) Global analysis of allele-specific expression in *Arabidopsis thaliana* . Genetics 182: 943–954. 19474198 10.1534/genetics.109.103499 PMC2728882

[17] Palmieri N, Nolte V, Suvorov A, Kosiol C, Schlotterer C (2012) Evaluation of different reference based annotation strategies using RNA-Seq – a case study in *Drososphila pseudoobscura* . PLoS One 7: e46415. 23056304 10.1371/journal.pone.0046415 PMC3463616

[18] Kofler R, Orozco-terWengel P, De Maio N, Pandey RV, Nolte V, et al (2011) PoPoolation: a toolbox for population genetic analysis of next generation sequencing data from pooled individuals. PLoS One 6: e15925. 21253599 10.1371/journal.pone.0015925 PMC3017084

[19] Wu TD, Nacu S (2010) Fast and SNP-tolerant detection of complex variants and splicing in short reads. Bioinformatics 26: 873–881. 20147302 10.1093/bioinformatics/btq057 PMC2844994

[20] Degner JF, Marioni JC, Pai AA, Pickrell JK, Nkadori E, et al (2009) Effect of read-mapping biases on detecting allele-specific expression from RNA-sequencing data. Bioinformatics 25: 3207–3212. 19808877 10.1093/bioinformatics/btp579 PMC2788925

[21] Pandey RV, Franssen SU, Futschik A, Schlotterer C (2013) Allelic imbalance metre (Allim), a new tool for measuring allele-specific gene expression with RNA-seq data. Mol Ecol Resour 13: 740–745. 23615333 10.1111/1755-0998.12110 PMC3739924

[22] Robinson MD, Oshlack A (2010) A scaling normalization method for differential expression analysis of RNA-seq data. Genome Biol 11: R25. 20196867 10.1186/gb-2010-11-3-r25 PMC2864565

[23] Landry CR, Wittkopp PJ, Taubes CH, Ranz JM, Clark AG, et al (2005) Compensatory *cis-trans* evolution and the dysregulation of gene expression in interspecific hybrids of *Drosophila* . Genetics 171: 1813–1822. 16143608 10.1534/genetics.105.047449 PMC1456106

[24] Berriz GF, Beaver JE, Cenik C, Tasan M, Roth FP (2009) Next generation software for functional trend analysis. Bioinformatics 25: 3043–3044. 19717575 10.1093/bioinformatics/btp498 PMC2800365

[25] Lemos B, Araripe LO, Fontanillas P, Hartl DL (2008) Dominance and the evolutionary accumulation of *cis*- and *trans*-effects on gene expression. Proc Natl Acad Sci U S A 105: 14471–14476. 18791071 10.1073/pnas.0805160105 PMC2567206

[26] Graze RM, McIntyre LM, Main BJ, Wayne ML, Nuzhdin SV (2009) Regulatory divergence in *Drosophila melanogaster* and *D. simulans*, a genomewide analysis of allele-specific expression. Genetics 183: 547–561, 541SI–521SI. 10.1534/genetics.109.105957 PMC2766316 19667135

[27] Wittkopp PJ (2005) Genomic sources of regulatory variation in *cis* and in *trans* . Cell Mol Life Sci 62: 1779–1783. 15968467 10.1007/s00018-005-5064-9 PMC11139219

[28] Wray GA (2007) The evolutionary significance of *cis*-regulatory mutations. Nat Rev Genet 8: 206–216. 17304246 10.1038/nrg2063

[29] Wittkopp PJ, Kalay G (2012) *Cis*-regulatory elements: molecular mechanisms and evolutionary processes underlying divergence. Nat Rev Genet 13: 59–69. 10.1038/nrg3095 22143240

[30] Bedford T, Hartl DL (2009) Optimization of gene expression by natural selection. Proc Natl Acad Sci U S A 106: 1133–1138. 19139403 10.1073/pnas.0812009106 PMC2633540

[31] Schaefke B, Emerson JJ, Wang TY, Lu MY, Hsieh LC, et al (2013) Inheritance of gene expression level and selective constraints on trans- and cis-regulatory changes in yeast. Mol Biol Evol 30: 2121–2133. 23793114 10.1093/molbev/mst114

[32] Marioni JC, Mason CE, Mane SM, Stephens M, Gilad Y (2008) RNA-seq: an assessment of technical reproducibility and comparison with gene expression arrays. Genome Res 18: 1509–1517. 18550803 10.1101/gr.079558.108 PMC2527709

