# Supplementary figures and images for: Intra-Specific Regulatory Variation in Drosophila pseudoobscura
Source: PLoS One. 2013 Dec 27;8(12):e83547. doi: 10.1371/journal.pone.0083547 (PMC3873948; doi:10.1371/journal.pone.0083547)

**A**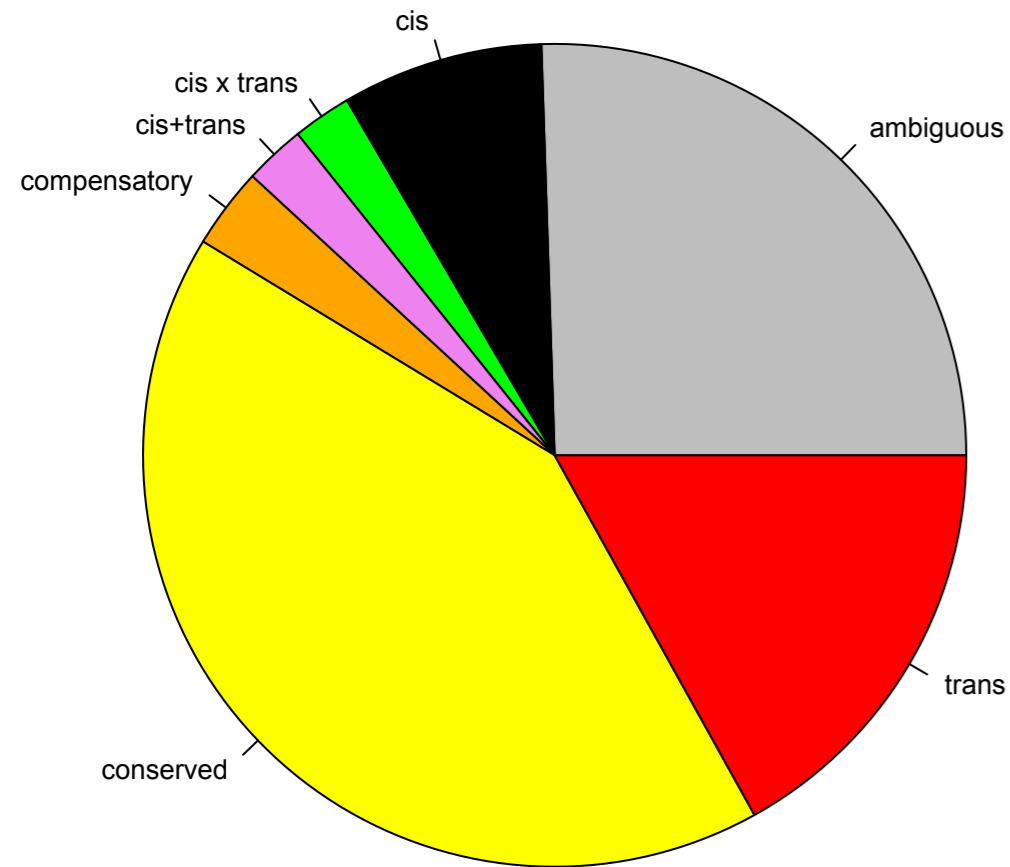**B**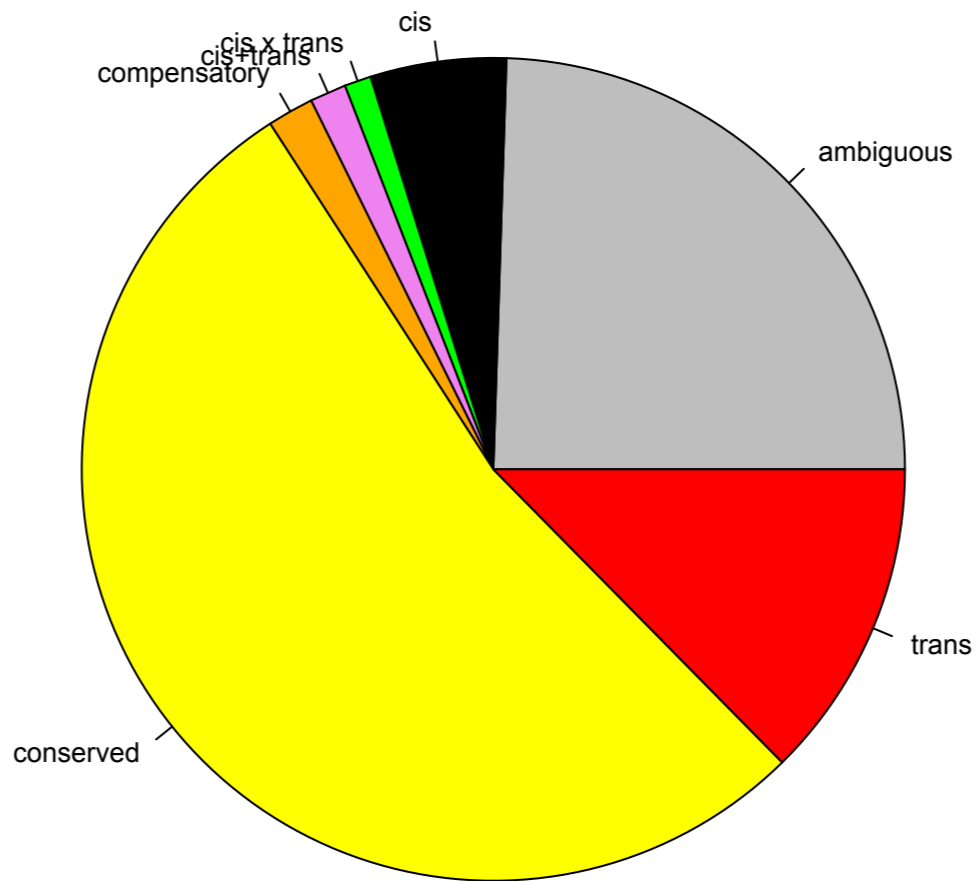**C**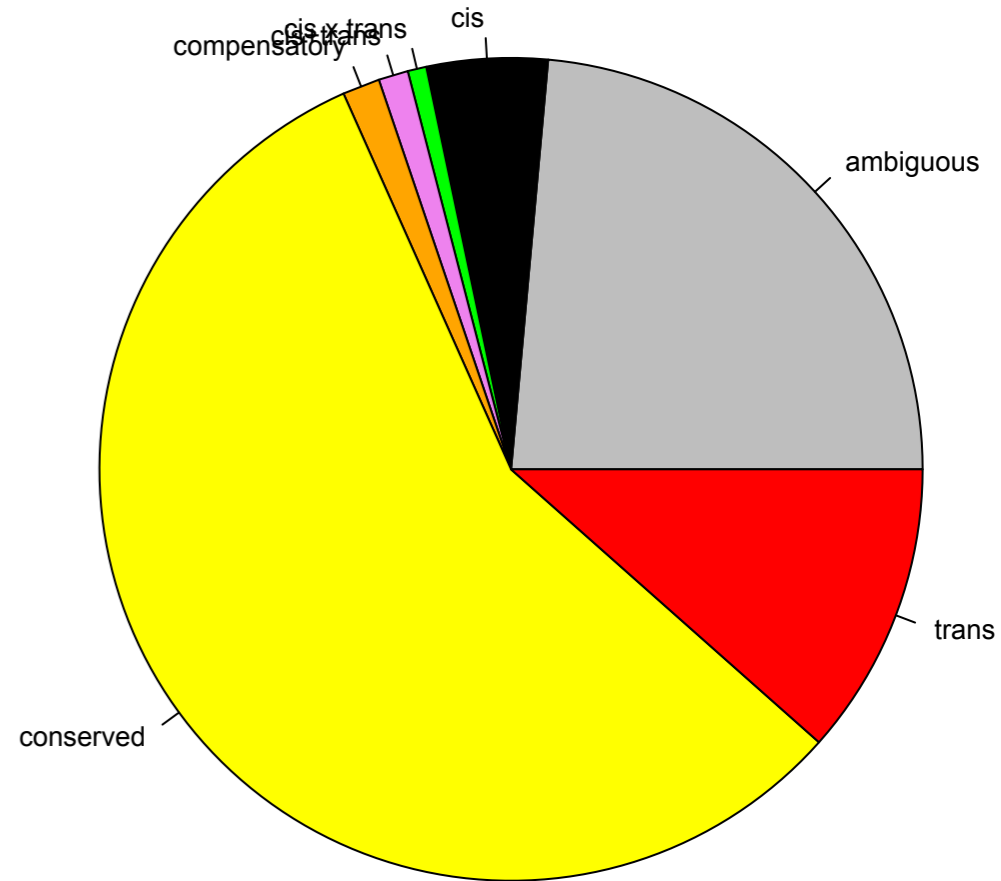

Supplement: Figure S1 — Effect of different FDR threshold on the identification of regulatory effects. A) FDR < 0.05. B) FDR < 0.01. C) FDR <0.005. (PDF) [file pone.0083547.s001.pdf]

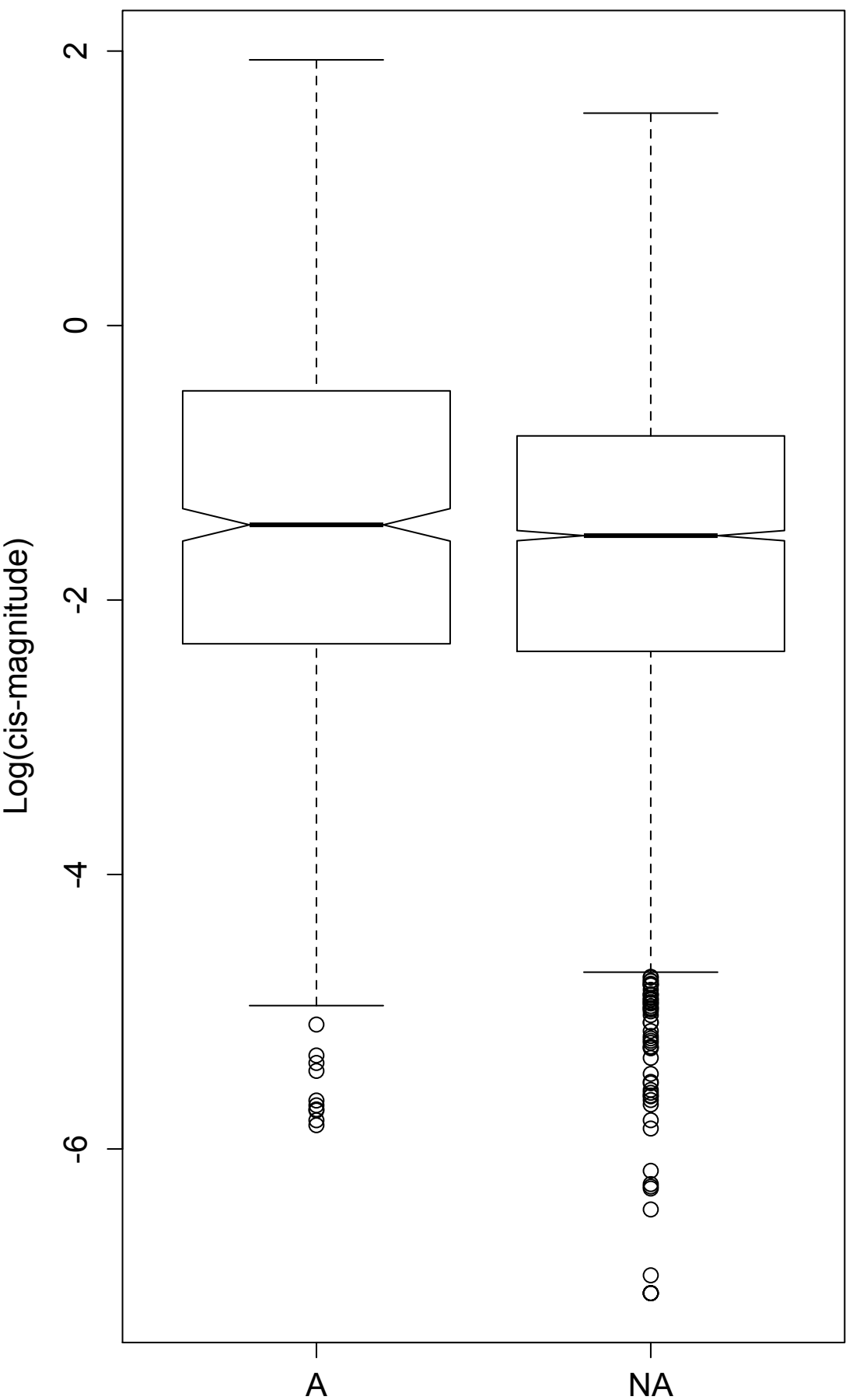

Supplement: Figure S2 — Cis -magnitude comparison of genes with additive and non-additive modes of inheritance. Genes with additive mode (A) of inheritance exhibit higher cis-magnitude vs. all other genes with non-additive modes (NA) excluding genes with conserved expression mode. (PDF) [file pone.0083547.s002.pdf]
