# Supplementary material for: Intra-Specific Regulatory Variation in Drosophila pseudoobscura
Source: PLoS One. 2013 Dec 27;8(12):e83547. doi: 10.1371/journal.pone.0083547 (PMC3873948; doi:10.1371/journal.pone.0083547)
Supplement: Table S1 — Determination of regulatory categories based on total differential expression between parents (TDE), allele-specific differential expression in F1 hybrid (cis) and trans-test. All the statistical tests were performed using G-test, followed by FDR correction (q≤0.05). (DOC) [file pone.0083547.s003.doc]

Table S1. Determination of regulatory categories based on total differential expression between parents (TDE), allele-specific differential expression in F1 hybrid (*cis*) and *trans*-test. All the statistical tests were performed using G-test, followed by FDR correction (q ≤ 0.05).

| **Category** | **TDE** | **cis** | **trans^2^** |
| --- | --- | --- | --- |
| cis | TRUE | TRUE | FALSE |
| trans | TRUE | FALSE | TRUE |
| cis+trans^1^  cis-by-trans | TRUE | TRUE | TRUE |
| Compensatory | FALSE | TRUE | TRUE |
| Conserved | FALSE | FALSE | FALSE |
| Ambiguous | FALSE | TRUE | FALSE |
| Ambiguous | FALSE | FALSE | TRUE |

^1^ According to the classification defined in .

If the expression of opposite alleles is favored (log2(ps88/ps94)*log2(ps88_hyb/ps94_hyb)<0) is classified as *cis*-by-*trans*. If the expression of the same alleles is favored (log2(ps88/ps94)*log2(ps88_hyb/ps94_hyb)>0) is classified as *cis*+*trans.*

^2^TDE=*cis*+*trans* , hence *trans* is significant only if TDE≠*cis*.

References

1. McManus CJ, Coolon JD, Duff MO, Eipper-Mains J, Graveley BR, et al. (2010) Regulatory divergence in *Drosophila* revealed by mRNA-seq. Genome Res 20: 816-825.

2. Wittkopp PJ, Haerum BK, Clark AG (2004) Evolutionary changes in *cis* and *trans* gene regulation. Nature 430: 85-88.
