# Supplementary material for: Intra-Specific Regulatory Variation in Drosophila pseudoobscura
Source: PLoS One. 2013 Dec 27;8(12):e83547. doi: 10.1371/journal.pone.0083547 (PMC3873948; doi:10.1371/journal.pone.0083547)
Supplement: Table S2 — Identification of inheritance modes based on differential expression and directionality of expression between parental and total expression in F1 hybrids. (DOC) [file pone.0083547.s004.doc]

Table S2. Identification of inheritance modes based on differential expression and directionality of expression between parental and total expression in F1 hybrids.

| **Inheritance mode** | **ps88≠hyb**^1^ | **log(ps88/hyb)<0** | **ps94≠hyb** | **log(ps94/hyb)<0** |
| --- | --- | --- | --- | --- |
| Additive | TRUE | FALSE | TRUE | TRUE |
| Additive | TRUE | TRUE | TRUE | FALSE |
| Over-dominant | TRUE | TRUE | TRUE | TRUE |
| Under-dominant | TRUE | FALSE | TRUE | FALSE |
| Dominant | FALSE | FALSE | TRUE | FALSE |
| Dominant | FALSE | TRUE | TRUE | FALSE |
| Dominant | FALSE | FALSE | TRUE | TRUE |
| Dominant | FALSE | TRUE | TRUE | TRUE |
| Dominant | TRUE | FALSE | FALSE | FALSE |
| Dominant | TRUE | TRUE | FALSE | FALSE |
| Dominant | TRUE | FALSE | FALSE | TRUE |
| Dominant | TRUE | TRUE | FALSE | TRUE |
| Conserved expression | FALSE | TRUE | FALSE | TRUE |
| Conserved expression | FALSE | FALSE | FALSE | FALSE |
| Conserved expression | FALSE | FALSE | FALSE | TRUE |
| Conserved expression | FALSE | TRUE | FALSE | FALSE |

^1^Using the G-test parental gene expression is compared with total hybrid (allele 1 +allele 2) expression. Due to multiple testing FDR correction was applied to the significance level of 0.05.
