# Supplementary material for: Intra-Specific Regulatory Variation in Drosophila pseudoobscura
Source: PLoS One. 2013 Dec 27;8(12):e83547. doi: 10.1371/journal.pone.0083547 (PMC3873948; doi:10.1371/journal.pone.0083547)
Supplement: Table S3 — Library size information. (DOC) [file pone.0083547.s005.doc]

Table S3. Library size information

| **Strain** | **ps88f** | **ps94f** | **ps94xps88f** |
| --- | --- | --- | --- |
| Total pairs | 42 394 057 | 42 265 943 | 40 327 663 |
| Number of reads after filtering | 4 197 294 | 4 162 996 | 3 859 202 |
